# Ohio Medical Convention

**Published:** 1849-08

**Authors:** 


### THE WESTERN JOURNAL.

THIRD SERIES.—VOL. IV.—NO. II.

LOUISVILLE, AUGUST 1, 1849.

### OHIO MEDICAL CONVENTION.

At the Convention of the Physicians of Ohio, held at Columbus, on the 5th of June, 1849, the following resolutions were adopted, having a bearing upon Medical Schools:

Resolved, That in the opinion of this Convention, it is inexpedient for the Medical Schools of Ohio, at this time, to lengthen the term of their sessions; but it should be strictly enjoined upon students to be present at the beginning, and to continue in attendance until the end of the session;

Which was amended by adding the following resolutions, by Dr. J. C. Norton:

Resolved, That the practice of admitting young men to graduate, by attending upon one course of lectures only, considering four years' practice as equivalent to attendance upon another, is unjust to our Medical Schools, and to the community, as it indirectly holds out inducements to young men to engage in the practice of medicine, without due qualifications.

Resolved, That the Professors of the Medical Schools in this State, be requested to confer together upon the subject, with a view of correcting this evil.

An attempt was made by the professors in the Botanical and Eclectic Medical Schools in Cincinnati, during the last session of the Ohio Legislature, to gain admission to the Commercial Hospital on equal terms with the professors in the Ohio Medical College. With great propriety the Senate rejected the proposition, and we are glad to find the Convention sustaining this wise and dignified action of the Legislature. The following resolutions were adopted on that subject:

Be it Resolved, by the Ohio State Medical Convention, now in session, That it is inexpedient to admit into the Commercial Hospital and Insane Asylum any other lecturers and practitioners than those now recognized by law as holding connection with that institution; as a different policy would directly embarrass its movements, disturb its harmony, and greatly lessen its usefulness.

Resolved, That the primary and most important object of the Hospital is to provide for, and administer to the necessities of the indigent and destitute, and but incidentally to add to the advantages of medical classes; and to the end that the humane and benevolent objects for which such institutions are established be fully realized, it is proper that they be held and maintained in a state of quietude and comfort, so far as the circumstances connected with the government can be made to effect it.

Resolved, That any other policy than that contemplated in the act of the Legislature establishing the said Commercial Hospital and Lunatic Asylum, would be subversive of the objects which dictated its establishment, and consequently subversive of any rights and privileges of those who, for many years, have borne the responsibilities, and performed the labors of said Commercial Hospital and Lunatic Asylum of Ohio, located at Cincinnati.

Resolved, That we recognize, in the acts of the last Legislature of Ohio upon this subject, evidence of that wisdom, humanity and justice, which originated and perfected the establishment of the institution.

A number of interesting papers were read to the Convention, some of which we have transferred to our pages.

